# Public and participant involvement as a pathway to inclusive dementia research

**DOI:** 10.1002/alz.14350

**Published:** 2024-11-14

**Authors:** Sarah Walter, RÍona McArdle, Emily A. Largent, Rebecca Edelmayer, Claire Sexton, Sandra Loyola Sandoval, Helen Medsger, Nancy Meserve, Roland Samaroo, Cynthia Sierra, Marlon M. P. Smeitink, Allison Gibson, Sarah Gregory, Diana Karamacoska, Iracema Leroi, Doris Molina‐Henry, Aida Suarez‐Gonzalez, Crystal M. Glover

**Affiliations:** ^1^ Alzheimer's Therapeutic Research Institute, University of Southern California San Diego California USA; ^2^ Newcastle University Newcastle upon Tyne UK; ^3^ University of Pennsylvania Perelman School of Medicine Philadelphia Pennsylvania USA; ^4^ Alzheimer's Association Chicago Illinois USA; ^5^ PPI Member San Diego California USA; ^6^ Saint Louis University School of Social Work St. Louis Missouri USA; ^7^ Edinburgh Dementia Prevention Centre for Clinical Brain Sciences University of Edinburgh Edinburgh UK; ^8^ NICM Health Research Institute Western Sydney University Penrith New South Wales Australia; ^9^ School of Medicine and Global Brain Health Institute Trinity College Dublin Dublin UK; ^10^ Dementia Research Centre UCL Queen Square Institute of Neurology London UK; ^11^ Rush Alzheimer's Disease Center Rush Medical College Chicago Illinois USA; ^12^ Department of Psychiatry and Behavioral Sciences Rush Medical College Chicago Illinois USA; ^13^ Department of Neurological Sciences Rush Medical College Chicago Illinois USA

**Keywords:** community engagement, person‐centered research, public and patient involvement

## Abstract

The field of Alzheimer's disease and related dementias (ADRD) urgently requires inclusive research to ensure the priorities and outcomes of research apply to those most impacted. We postulate public and participant involvement (PPI) as a pathway to achieving the best science, both in research that informs health and social policy as well as in therapeutic studies to treat and prevent ADRD. This position paper aims to provide dementia researchers with evidence to understand how to apply PPI. We begin by highlighting the disparities experienced by people with dementia, including ageism, stigma of cognitive impairment, and health disparities for minoritized communities. We then provide examples of PPI in ADRD across the research lifecycle, from defining research topics of priority to those impacted by ADRD, through the design, analysis, dissemination, and translation to policy and practice. We also provide recommendations to create and maintain collaboration between researchers and communities through PPI.

**Highlights:**

- A central premise of public and participant involvement (PPI) is collaborative relationships between researchers and community members.
- To build equitable partnerships, researchers must acknowledge and understand the context of research. This includes ageism, the stigma of dementia, and ongoing discrimination for many minoritized communities.
- Meaningful partnerships include choice, respect, shared decision making, access, inclusion, and representation. Notably, we recommend that researchers begin partnerships early in the research process and share the impact of PPI on research.

## 1 INTRODUCTION

Alzheimer's disease (AD) and related dementias (ADRD) represent an urgent health need with a growing global impact as the number of people living with ADRD is expected to increase to 155 million by 2050.^1^, ^2^ People living with dementia exist within a widening chasm of heath disparities, particularly for individuals who have been minoritized due to race, ethnicity, immigration status, gender and sexual identity, religion, and socio‐economic status. Currently, it is largely unknown how and what people affected by ADRD think about aging and dementia and the role research can play in alleviating burdens associated with ADRD. Without insights from those with lived experience, the research that informs our knowledge—from psychosocial and resilience factors to biomarker and drug discovery—will remain incomplete and inequitable.

In general, research studies do not critically examine or incorporate perspectives of people living with an ADRD diagnosis, those at higher risk for developing ADRD, or their support partners. Following classic research methodologies that emphasize separation between researchers and participants to minimize bias (i.e., positivism^3^), it is common for researchers to design a study, obtain necessary funding, and begin to recruit study participants—all without acknowledging and understanding prospective participants’ lived experience. The lack of these perspectives become glaringly evident once study challenges occur, such as slow recruitment, poor retention, and lack of representation among those enrolled.^4^, ^5^ Here, we argue that public and participant involvement (PPI) will not only address practical challenges^6^ but also inform a fuller understanding of ADRD, and to ensure relevance of research results.

PPI is broadly defined as “conducting research with or by the public; rather than to, about, or for them.”^7^, ^8^ PPI involves a collaborative relationship, rather than only interacting with participants to collect data. Effective use of PPI improves the relevance and quality of research by incorporating the perspectives and lived experiences of those affected.^7^, ^9^ Partnerships between researchers and participants, built through PPI, can ensure that research findings are meaningful and serve those individuals and communities most impacted by dementia. People with lived experience of ADRD have expressed their willingness to contribute but uptake by researchers has been sparse.^10^, ^11^, ^12^ When PPI is used in ADRD research, a key limitation has been the lack of representativeness among the patients and members of the public included. We postulate that PPI should be a central tenet of ADRD research, and that use of PPI can enable researchers to actively work toward health equity^13^, ^14^ by intentionally partnering with communities who have been underserved by and underincluded in ADRD research.^15^, ^16^

## 2 PURPOSE

This position paper aims to equip ADRD researchers—from bench scientists to trialists, from epidemiologists to care researchers—with the need for and application of PPI. Frameworks for research partnerships using PPI are varied, with multiple models demonstrating meaningful impact.^17^ Our aim is to empower researchers to actively develop a framework to best serve their research as well as the communities in which they partner. To do so, we first outline the marginalization of persons living with dementia which underlies where research occurs. We then discuss how researchers can acknowledge these disparities, include PPI, and develop and sustain community partnership. We provide examples of effective PPI across all stages of research—from conceptualization to dissemination and translation. Finally, we discuss key considerations for inclusive ADRD research.

## 3 METHODS

This position paper was led by the Partnering with Research Participants Professional Interest Areas (PIAs) of the Alzheimer's Association International Society to Advance Alzheimer's Research and Treatment (ISTAART; alz.org/ISTAART). Co‐authors include professional researchers together with five community members from five countries (the Netherlands, Ireland, United Kingdom, Australia, and the United States) with lived experience of ADRD and PPI research. Co‐authors drafted and reviewed manuscript drafts by e‐mail, including three video conference meetings with community members to discuss the paper's emphasis and approach. All co‐authors provided insights on terminology and inclusion of lived experience. We have included direct quotes (in italics) from Partnering with Research Participants PIA community members to amplify their voices and illustrate their expertise. A lay summary of this paper has also been co‐developed to be disseminated to community members.

Foundational to this position paper was a collaborative discussion and consensus on key terminology. The following are key terms and how we have agreed to use them. Co‐authors aimed for terms to acknowledge systematic exclusion, offer opportunities for inclusion, and to be globally relevant.

- *Public and Participant Involvement (PPI)* is an umbrella term for inclusion of diverse perspectives and lived experiences in the research process. PPI is also referred to as public and patient involvement;^18^ however, as ADRD research includes non‐patient partners such as individuals living with elevated risk^19^ without cognitive impairment and support partners, “participant” is viewed as more inclusive.
- *Researcher* refers to individuals who professionally conduct and engage in research, not limited to faculty members or principal investigators.
- *Person living with or affected by dementia* refers to individuals diagnosed with or at risk for ADRD. This person‐first language emphasizes the individual over their diagnosis or disease risk.
- *Support partner* refers to those who assist and provide care for a person living with dementia.
- *PPI member* refers to a member of the public, a person living with or affected by dementia, or a support partner who engages with researchers in PPI.
- *Marginalized communities* refers to groups that are underserved or underengaged in research and health‐care settings due to any or intersections of the following: age, sex, race, ethnicity, country of origin, religion, socioeconomic status, geographic location, sexual and gender identities, and disability status.^20^, ^21^ Here we define community as “a group of people with diverse characteristics who are linked by social ties, share common perspectives, and engage in joint action in geographical locations or settings.”^22^

## 4 MARGINALIZATION OF PERSONS LIVING WITH DEMENTIA

Individuals living with dementia experience social exclusion on multiple levels, including interpersonal relationships, local communities, and more broadly in the institutions and systems of social and health care. Ageism contributes to this marginalization for a majority of those living with dementia.^23^ Intersecting with ageism is the stigma of a dementia diagnosis. Fear of stigma may lead individuals and support partners to underreport symptoms and delay seeking a diagnosis, and clinicians not communicating a diagnosis to individuals and families.^24^ As stigma is rooted in lack of knowledge, the evolving definition of AD^25^ may lead to greater confusion and more stigma.^26^, ^27^ People living with dementia have pushed back against the dehumanizing impact of stigma with a simple but powerful statement; *“I am still here.”* ^28^, ^29^, ^30^

Ageism and dementia stigma are compounded for individuals who have experienced life‐long discrimination due to their race, ethnicity, gender identity, ability, and socioeconomic status.^31^ Evidence of this impact includes lower rates of diagnosis, poor social support, low quality of care, and higher financial burden for minoritized communities.^13^, ^24^, ^32^, ^33^, ^34^, ^35^ Work done in low and middle income countries has shown that PPI can have broader impact on communities, including changing attitudes about research and dementia.^36^ The marginalization of individuals with learning disabilities must also be considered. Adults with Down syndrome have a higher than 80% risk of developing AD by their mid‐50s.^25^, ^37^, ^38^ These health disparities are upheld by systems and are well documented globally.^24^, ^39^

We set forth that marginalization of people with dementia compounds with other causes for health disparities and is not an anomaly but the current state of health care and related research. Decisions to engage in research are both specific to the individual and deeply rooted in the broader context.^13^ Before engaging in PPI, researchers must first actively educate themselves and acknowledge the structures that underpin disparities, including both historical and ongoing unethical research practices.^40^ Researchers, whatever their personal ethos, are representatives of medical and academic systems that actively and passively perpetuate inequity, injustice, and racism.^33^ These systems have deleteriously impacted the very communities in which researchers most require partnerships to design inclusive research.

Hence, it is critical that researchers approach communities with the primary aim to learn about these groups.^41^ Researchers must begin by asking community members, “What are your needs? What are the biggest issues impacting your health and your community?” These discussions must be bidirectional, and researchers must be prepared to transparently respond to questions. One of our community member co‐authors explained, “*Community members may ask of researchers, ‘Why should I trust you? You need to establish a relationship; a dialogue; I getting to know you and you getting to know me.’”*

## 5 PUBLIC AND PATIENT INVOLVEMENT THROUGHOUT THE RESEARCH PROCESS

**FIGURE 1:**
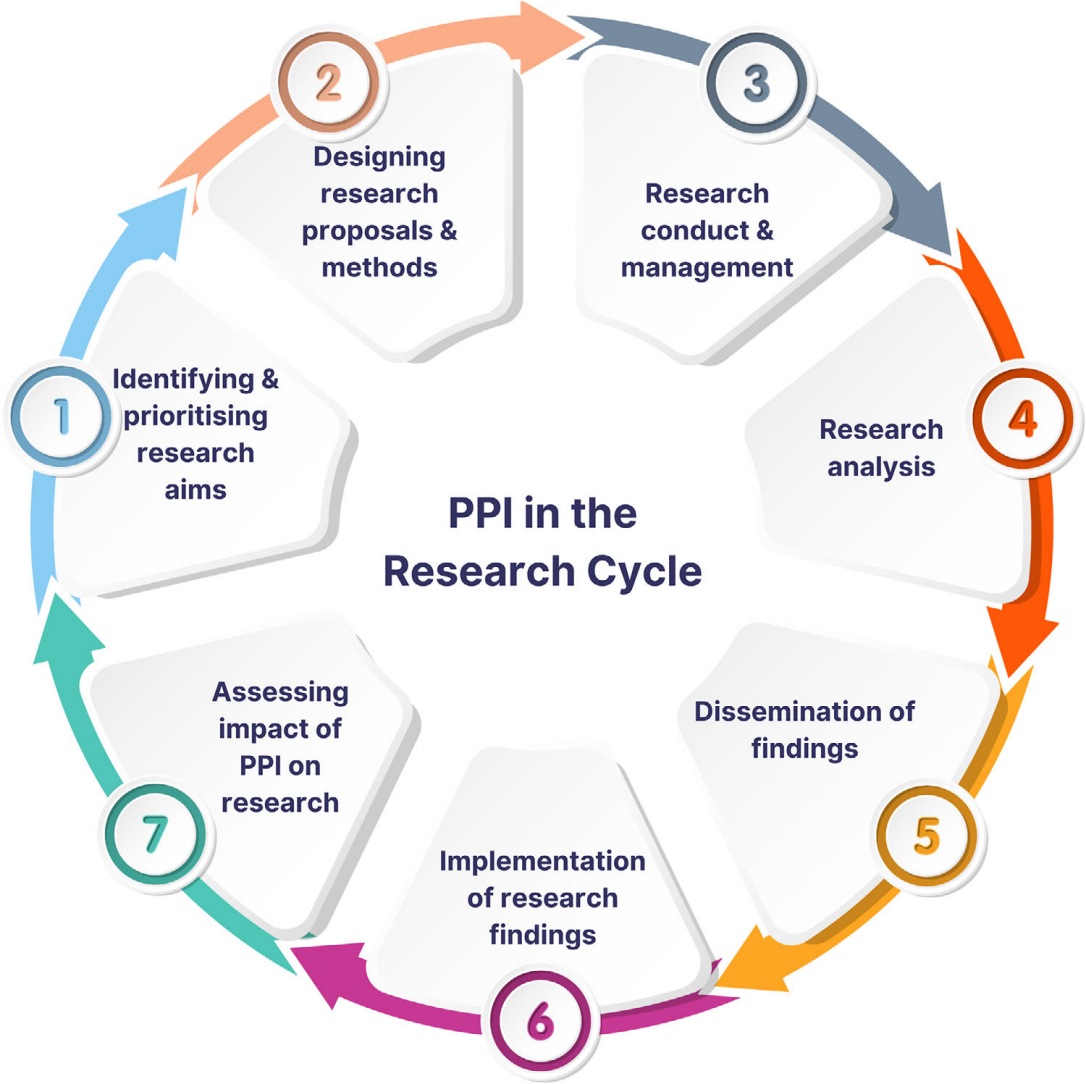
Public and participant involvement (PPI) in the research cycle. Modified from the UK National Institute on Health Research^8^

Effective use of PPI adds value to every step of research. We turn now to describe examples and review approaches throughout the research cycle, as illustrated in Figure 1.

### Step 1. Identifying and prioritizing research topics and aims

Individuals living with dementia have espoused the desire to be involved in research from inception.^12^, ^42^ Many funding organizations now require that researchers demonstrate PPI in the development of the research question and initial design.^43^, ^44^, ^45^ Identifying and prioritizing research topics requires a bi‐directional exchange of knowledge—combining empirically identified researchers’ priority areas with PPI members’ perspectives.^17^, ^42^, ^46^ This is an iterative process, and may not always lead to consensus; differences should be acknowledged with mutual respect and decisions should be communicated transparently.^47^ PPI members can challenge assumptions held by researchers and bring pragmatic questions and solutions, leading to improved clarity and accessibility.^47^ For example, the research questions of the ActivDem study were co‐designed by people living with dementia, who emphasized the role of their support partners in facilitating physical activity (Table 1). This collaboration resulted in research questions and methodology adapted to include an exploration of support partners’ psychosocial well‐being and care management strategies.^48^

**TABLE 1:** ActivDem research aims prior to and after public and patient involvement (PPI).

Initial research aims	PPI‐refined research aims
Identify modifiable influences of physical activity in people living with dementia.	Identify psychosocial characteristics in people living with dementia and their support partners which predict habitual physical activity change in dementia.
Examine whether changes in physical activity can help predict who will lose their independence faster, and if staying physically active can slow this down.	Identify how social support and health care nationalize impact habitual physical activity change in dementia. Examine whether decline in habitual physical activity reflects decline in functional independence in dementia.

Practical support of PPI members is essential to facilitate co‐development and refinement of research questions. Recommendations include providing a group with peers, adequate time to prepare, providing a pre‐meeting to allow time to digest issues and study, as well as a time to answer questions either as a group or one to one.^47^, ^49^ Safe spaces must be provided to allow PPI members the freedom to express opinions and perspectives.^47^ Meaningful results will depend on whether researchers are able to build trusting relationships and provide flexibility in engagement options.^50^ *“The design of research questions built on patient and public involvement in a partnership of mutual trust, shared expertise and the lived experience of those with ADRD and their care partners,”* one community member co‐author said, *“is likely to significantly advance the feasibility, validity and applicability of such research.”*

### Step 2. Designing research proposals and methods

PPI during the proposal development stage can ensure that research is undertaken in a meaningful and targeted manner, using culturally competent methods. Ideally, this would include PPI members as co‐applicants and members of the study team. PPI advisory boards have also been established, guiding research design, conduct of studies, governance, and management.^51^ Feedback from these groups has emphasized the importance of sharing individual research results (i.e., cognitive scores, magnetic resonance imaging results, genetic or amyloid results), with guidance on how sensitive results can be shared.^52^, ^53^ The input of PPI on study methods can ensure meaningful outcome measures are used.^36^ A pan‐European consultation found that people living with dementia want to participate in interventions focused on outcomes such as well‐being, social participation, and human rights, highlighting a need for significant improvements in research focusing on these meaningful outcomes.^54^ In the UK, a National Institute for Health Research project combined a literature review with expert consensus and PPI input to identify core outcome measures in disease‐modifying trials which are meaningful to patients.^55^ In the United States, the National Institute on Aging's Imbedded Pragmatic AD and ADRD Clinical Trials Collaboratory Lived Experience Panel was formed to advise the development of pragmatic clinical trials. Their feedback emphasized the importance of personhood and relationship over logistics.^56^

Prior PPI work has resulted in both practical guidance,^57^ problem solving and development of communication tools,^58^ and innovative methods to engage.^59^ A recent review identified community outreach as an ADRD research recruitment and retention strategy to engage minoritized communities, which could be bolstered using community‐led initiatives.^13^ One successful example was the co‐design of a culturally informed storytelling campaign for research with PPI members from the Black community, which was well received by community members.^60^

### Step 3. Research conduct and management

Research studies in ADRD oftentimes take > 1 year to conduct, necessitating research that responds to a changing context. A key example of the benefits of PPI in this stage is the support a PPI advisory group can bring to mitigate study recruitment challenges. PPI members can problem solve and suggest initiatives to enhance recruitment, including reviewing study materials, connecting the researchers with community leaders, or suggesting feasible alterations to the eligibility criteria. Study procedures may need to be adjusted to accommodate newly emerging tools, like blood‐based biomarkers, or newly approved medications. A PPI advisory group can play an important role in developing participant‐facing information, including the presentation of risks and unknowns regarding drug trials and other pharmacological or invasive therapies in an understandable manner to support true informed consent. Researchers can work with PPI members to determine what compensation for research participation would be appropriate, and what incentives should be offered to express gratitude to research participants. Depending on the needs and interests of the community, reimbursement and incentives may be both financial and non‐monetary (e.g., sharing individual research results or having access to high‐quality information).^61^ PPI members can also be trained to assist with data collection. Crowd‐sourced data collection or “citizen science” was used for the first time in an AD research project called “Stallcatchers,” the online participation of volunteers on the online game reduced the time required to analyze data.^62^

### Step 4. Research analyses

Here, data analyses are most effective when PPI members have input on design, outcomes, and conduct. Offering community members training and context for analysis will ensure equal contributions and the best‐informed collaborative decisions. In this step, it is the researcher's role to use their methods (whether quantitative or qualitative) to conduct the analysis and outline the results and to offer an interpretation of what the results mean. It is the PPI member's role to validate or disagree with that interpretation and ideally, to discuss and co‐develop an interpretation. PPI in analysis may help to focus the researcher on what is meaningful to people with lived experience, while avoiding erroneous assumptions about the meaning of results, especially when situational or cultural context is needed. Secondary data analysis, which leverages data collected for other purposes (e.g., transcripts from lived‐experience interviews or health‐care datasets) when analyzed in partnership with PPI, could allow for better use of data and expanded understanding of the results.^63^ In a meta‐ethnography of studies of walking, PPI was a critical piece of the analysis and development of a conceptual framework.^64^ An additional benefit of using participatory analysis approaches is the outcome of more inclusive data visualization,^65^, ^66^ which could enhance understanding of both professional and general audiences when disseminating research findings.

### Step 5. Dissemination of findings

Researchers are required to publish outcomes in scientific journals and present at scientific conferences to fulfill funding requirements and maintain their academic positions and typically lead dissemination of research results. Co‐authorship of scientific publications is an under‐used method that can ensure the voices and perspectives of PPI members who contributed to new learnings are heard in unison with the voice of the researcher. One example is a call for inclusive design of indoor/outdoor spaces for people living with a diagnosis of dementia, emphasizing citizenship and human rights.^67^ Co‐authorship of scientific papers can lead to a call for change in research culture, as seen in a recent example in which research participants initiated a bill of rights for sharing individual results in research.^68^ Researchers should discuss with PPI members the information the community needs and how to best disseminate new learnings. Additionally, journals can provide an open access community briefing section that distills the findings in an accessible way. A community member co‐author stated, *“Having the opportunity to be informed and knowledgeable to share within your community is powerful. It allows for discussions to be had in everyday life and engage others in what you are learning. Which may in return spark an interest in someone else to take part in research.”*

PPI in scientific conferences is not commonly practiced,^69^ either in ADRD or other specialty fields, with few published instances.^70^, ^71^ A conference program that included research participants in ADRD scientific conferences found that community members prefer full, unfiltered access to scientific content, and discovered inspiration in being included in scientific discussions.^11^ PPI doesn't have to be limited to listening. Co‐presentation is another PPI avenue that ensures recognition of PPI members in the research process and allows for lived experience to be included in the discussion, providing a richer context for the audience.^66^ Partnerships can be built in these forums, as in the case of this paper when co‐authors met during a session for the Alzheimer's Association International Conference (AAIC).

Dissemination of research findings can extend beyond scientific conferences and publications to public engagement events, blogs, webinars, and other media efforts. For example, sharing personal experiences was the primary motivator for Asian Americans and Pacific Islanders (AAPI) joining the Collaborative Approach for AAPI Research and Education (CARE) registry for dementia‐related research.^72^ Storytelling is a powerful tool for promoting more positive messages about dementia and the research experience. This has become the central tenet of nationalized, citizen‐led recruitment services such as Join Dementia Research in the UK,^73^ and StepUp for Dementia Research in Australia.^74^ Exemplifying that lived experiences and stories shared are not the property of researchers, some researchers have partnered with their participants to develop short films to share the stories of PPI members,^75^ create public‐facing handbooks, and conduct public webinars.^65^, ^66^

### Step 6. Implementation of findings

The implementation of findings sits at the intersection of clinical research and clinical care. It is this interface that communication with participants must be well planned and well executed to continue to develop trust in the research process and to provide value to the participants and the public. Incorporating lived experience of participants in clinical trials, including those that ended abruptly, can improve communications with research participants and care partners, providing information and support for next steps. The Participant Follow‐Up Improvement in Research Studies and Trials workgroup brought together representatives from academic, industry, government, and non‐profit AD/ADRD research communities, along with research participants and study partners, to establish recommendations for improving communication when trials do not end as planned. The 17 recommendations developed by the workgroup are not disease specific and could be used to improve communication and build partnership with research participants and study partners broadly.^76^, ^77^ These are the first recommendations of their kind developed for any therapeutic disease area and are already being implemented by researchers in industry (e.g., GRADUATE studies—gantenerumab topline results released^78^) and academia (e.g., A4 study—solanezumab topline results released^79^).

Community members’ perspectives are critical in understanding what will be most meaningful in terms of potential benefits or risks assumed when taking next steps to transition and implement new diagnostic, therapeutic, or care approaches in real‐world clinical practice. Personal perspectives on what it means to live with ADRD and how access to new treatments or diagnostics impacts a family can be one of the most influential reasons cited by government agencies and policy makers in their decision‐making process.^80^ Committee or advisory roles in community, national, or international consortia and councils create opportunities for inclusive PPI in ADRD research, policy, and care. As examples, persons living with dementia and support partners have participated in the Advisory Council on Alzheimer's Research, Care, and Services for the National Alzheimer's Project Act (NAPA), the Food and Drug Administration's (FDA) Patient Representative Program, the World Dementia Council, and in patient advocacy leadership roles.^80^ It is also encouraging to see a growing movement within the scientific and medical community in which PPI is being incorporated into development of clinical decision‐making tools, and practice guidelines to improve the clinical experience for patients and families.

### Step 7. Assessing impact of PPI

A final and often overlooked stage of PPI is measuring the impact of PPI on the research process, outcomes, and individuals involved. As there is no single best way to measure impact of PPI, we recommend that researchers evaluate and document the impact on the research, researchers, and PPI members as the project evolves.^66^, ^81^ This includes documenting the recommendations made by PPI members, the changes made to the project, and what outcomes were observed. Multiple assessment tools have been designed for use, which can be adapted to suit the needs of the research project or program.^82^ There may also be significant and unmeasured impact of partnership and how it changes the perceptions and approach of researchers and PPI members such as shared purpose, passion, and being part of something larger than oneself. ​ When asked how they would know PPI was successful, a community member co‐author explained, *“The researcher has clearly adapted various aspects of the study and before funding applications to take into account the experiences and views of the PPI group members. I also feel that it helps the researcher to talk to us about the study, to share the problems and feel good about the successes.”*

## 6 KEY CONSIDERATIONS

When engaging in PPI, investigators must be aware of a few key considerations, which, if not considered in partnership with community members, can impact the conduct, quality, and outcomes of PPI research. We discuss four key considerations to address these challenges. We also summarize recommendations to support meaningful PPI engagement (Table 2).

**TABLE 2:** Recommendations for public and participant involvement (PPI) in Alzheimer's disease and related dementias research.

**Establish a partnership with choice and shared decision making**. Ground partnership by valuing the needs, lived experience, and expertise of the community participants.
**Ensure equal access**. Ask for input on how to make interactions effective and accessible, including how group is structured, and settings for interactions.
**Start early, but it's never too late to engage**. PPI input can influence the focus and priorities of research, so it's best to start partnerships early in the process of research.
**Ensure representation and respect differences**. Be intentional in composing your group, ensuring voices not commonly heard are given space. Avoid single representatives or scenarios in which PPI are outnumbered by researchers.
**Discuss and define terms, titles, and roles**. The language we use demonstrates partnership, discuss with the community, and avoid acronyms and “academic speak.”
**Encourage storytelling**. Storytelling is a legitimate method of sharing wisdom, and patient stories are an important piece of evidence to inform care.
**Share impact**. Summarize for both community and researchers the impact of PPI on research and involve PPI in dissemination of learnings to both general audiences and scientific spaces.
**Demonstrate appreciation**. Ask what incentives would best show appreciation, and would be meaningful and helpful, such as a financial honorarium or a certificate or public acknowledgement; this will differ across communities.^96^
**Offer training to support co‐learning**. Training can benefit and empower both PPI members and researchers, but make sure training is not a barrier to participation.
**Listen and keep listening**. Feedback from PPI will clarify understanding on the part of both researchers and community.
**Respect confidentiality and privacy**. Assure PPI that their input will be kept confidential and anonymous.
**Demonstrate long‐term commitment**. Make long‐term plans, work toward short‐term goals, plan together with community.

Researchers should strive to understand the global, national, and local context in which research will take place and set up steps to include marginalized communities in these research partnerships. PPI members, including those living with a diagnosis and their support partners, should be brought together with people working in professional dementia services (e.g., clinicians, care professionals) and those with expertise in specific areas (e.g., researchers, technical experts).^46^, ^83^ A careful balance between community members and those with knowledge and/or power must be sought to allow for a true participatory effort, avoiding single PPI representatives.^84^ It is through PPI that the distinct expertise of community members and researchers can be brought together to advance our understanding of dementia.

### 6.1 Communication

Communication is foundational to effective PPI, and must be thoughtfully approached. To prepare themselves for inclusive communication, researchers should consider undergoing cultural sensitivity training.^51^ Community members may reference their personal stories in research‐related discussions. Storytelling is a culturally and linguistically respected means of sharing wisdom and beliefs. Although some health‐care research acknowledges the value of patient stories as evidence,^85^ researchers should be prepared to listen and understand stories, to incorporate their value into the research process. Also key to preparation is setting up the partnership as co‐learning, with researchers receiving training from community members on what is of value and priority to the community. Early in the process of engaging with community, researchers should ask PPI members if providing training to them (e.g., research ethics, methods, and terminology) would enhance comfort or be of personal value.^86^ Researchers should ask the preferred methods to communicate and offer multiple methods on a schedule that is best for community. Written materials should be provided both before and after any in person/video interactions. Researchers must communicate what is and what isn't within the scope of PPI and the project so participants understand what is reasonable to accomplish. “*The researcher needs to be clear what is required,”* one community member explained. *“It needs to be made clear to contributors that whilst they will be contributing their experience, it's not all about them…*. *It is also important that the researcher does what they say they will do and that they keep in touch even if it's just a short ‘catch up’ e‐mail between e‐mails.”*

Throughout the relationship, researchers should avoid the use of acronyms, jargon, technical terms, and academic language that may be unfamiliar to community members. One guide for dementia‐friendly language, developed by people with lived experience for the Global Brain Health Institute (GBHI), recommends use of terms that are centered on personhood that are culturally appropriate.^87^ The Canadian Consortium on Neurodegeneration in Aging (CCNA) co‐developed tips for involvement of people with lived experience of ADRD in meetings.^88^ Before, during, and after research, investigators must query PPI members to ensure that their message is being received and understood in the way it was intended. Meaningful and consistent communication will ground the partnership. *“There is a lot of communication in a working PPI model.”*

### 6.2 Personal and shared trauma and stigma of living with dementia

Participating in PPI requires considerable time and both emotional and cognitive investment. A person living with a diagnosis of dementia is facing a fatal disease. For many support partners, taking part in PPI means less time with their loved one. PPI members may also be taking part in a research study, which can add burden. These physical and emotional investments must be acknowledged and honored for what they are: a gift provided by someone with finite time.

Persons living with or at elevated risk of ADRD are aware of the significant public stigma of ADRD as well as the potential for discrimination in myriad settings.^89^ This stigma compounds disparate treatment due to ageism and may impact willingness to engage in research.^27^, ^90^ *“I don't think we've thought about ageism in studies. Ageism itself is a stigma, and a diagnosis adds to that.”* Hence, persons with a diagnosis or increased risk for ADRD may feel uncomfortable sharing their lived experience.

Researchers can demonstrate respect for communities by acknowledging the compounding impacts of dementia stigma for groups that have also experienced lifelong discrimination.^91^ This intersectionality has been highlighted by persons who have identified as sexual and gender minorities^92^ and people with Down syndrome.^93^ Speaking to this point, one community member co‐author explained, *“To not ask is to not see us.”* Another said, *“Everyone is facing different challenges in dementia, but there is some common ground. And by getting all those different voices in your PPI member group, you can make steps in dementia research!”*

### 6.3 Institutional requirements

Researchers operate within their medical and academic institutions and must abide by specific ethical and regulatory requirements as well as within funding parameters. If adequate time or support is not dedicated for PPI, researchers will be unable to develop necessary, meaningful relationships with community members, especially important when partnering with minoritized communities. Hence, it is incumbent upon researchers to plan adequately for the effort and time required, and work with their funders and home institutions to ensure planning, sufficient funding, and effort is built in to support long‐term community partnerships. Delayed reimbursement for PPI members paying up front for events or food can be costly to developing trust. A community member co‐author elaborated, “*Whilst no one does PPI work to make money, some recompense for time given is good practice. However, if the university or other organization is poor at paying expenses in a timely manner, this can reflect badly on the project.”*

Ethics boards may present barriers to building relationships with communities and should be considered by researchers early in planning.^65^, ^94^ Some countries require ethics review before engaging with community members, which limits ability of community members to provide early input. Additional ethical review may be requested to adequately remunerate PPI members for their time. Researchers can address these issues up front by engaging with institutional research offices, funding program officers, and communities prior to proposal submission; defining the inclusion of community engagement as an explicated and funded aspect of the research process; and defining PPI as community engagement necessary for equitable study design, research conduct, and maximum scientific impact for regulatory and ethics review boards.

### 6.4 Conflicting understandings, experiences, and priorities

Persons who decide to engage in research are diverse in their ADRD experiences. Meaning, a person living with a diagnosis, a person living with elevated risk, and a person providing support may all have separate and distinct motivators to engage in PPI that may conflict with each other. In addition, ADRD research has been largely limited in generalizability and applicability due to its ongoing exclusion of globally diverse groups.^95^ The largest increases of people with ADRD are projected to occur in lower‐ and middle‐income countries, where medical care is limited, most people with dementia never receive a diagnosis, and a majority of care is provided by unpaid caregivers who are mostly women and families.^96^ Use of PPI that includes and balances these diverse perspectives will ensure priorities for research are determined by local communities, not externally imposed.^36^ Researchers must acknowledge and note these different experiences and priorities to sustain meaningful PPI partnership, working within what people are able to do, and providing flexibility in levels of involvement; that is, working collaboratively with PPI members to reach a common goal that will serve both science and lived experiences. Furthermore, cognitive impairment may require adaptations to communication and reduce comfort in group discussions, which can be managed by offering one‐one‐one interactions or smaller groups. Additionally, there are different cultural understandings of dementia. For example, Indigenous people in North America see dementia as a natural part of the lifecycle, impacted by physiological, environmental, and psychological factors.^97^ Thus, communication between researchers and community members regarding individual beliefs, values, and preferences around dementia and research is empowering and builds trust. *“As someone that is caring for my mother, and actively in research, finding a drug would be great, but my goal is figuring out what is best for her well‐being, and how can she have a fulfilling rest of life while battling this illness, and how can we, her family, be supported to provide the care she needs.”*

## 7 DISCUSSION

By harnessing diverse perspectives and lived experiences of persons living with and affected by dementia and support partners, PPI can help the field of ADRD. PPI can advance equity by getting input needed to conduct research that is relevant and acceptable to diverse communities, inform recruitment and retention strategies to overcome lack of representation, confirm the validity of data interpretation, and disseminate findings for maximum impact. Researchers must be intentional in developing meaningful partnerships with minoritized communities. The examples highlighted in this paper illustrate that meaningful understanding of lived experience of dementia is not only feasible but offers clear benefits to dementia research.

While this position paper sets forth a conceptual and applied framework for PPI within ADRD research, there are several limitations. Although this paper was a collaboration between researchers and community members, we recognize that the audience for this paper is researchers; and we included a community briefing (see supporting information) to ensure our learnings can be disseminated more broadly. This paper did not include the perspectives of people with moderate or severe dementia, a group rarely included in PPI due to cognitive and functional challenges. Although one strength of the paper is the representation from individuals from different countries, it does not include authors from lower‐ or middle‐income countries. Standardized terminology is required to fully situate PPI in ADRD research and subsequent literature. The co‐authors partnered to set forth nomenclature for use in this paper, but these terms should be compared to and understood within local, national, and global environments.

## 8 CONCLUSION

The inclusion of PPI in research should be the standard. Substantial investments in resources, relationship building, funding, and time to cultivate is required. Our shared goal for ADRD research is best captured by the perspective of a community member co‐author: *“We want to see ADRD research accelerate and provide meaningful benefits to their lives, the lives of their loved ones, their children, and their communities. We seek to be seen, valued, and treated always as a person first.”*

## CONFLICT OF INTEREST STATEMENT

RM is funded by the National Institute for Health and Care Research (NIHR) for her fellowship (NIHR 301677) and supported by the NIHR Newcastle Biomedical Research Centre (BRC) based at The Newcastle upon Tyne Hospital NHS Foundation Trust, Newcastle University and the Cumbria, Northumberland and Tyne and Wear (CNTW) NHS Foundation Trust. SW receives funding from a grant for the Alzheimer's Clinical Trials Consortium (ACTC) National Institute on Aging (NIA), National Institutes of Health (NIH; U24AG057437). EL is supported in part by the National Institute on Aging (NIA) of the National Institutes of Health under Award Number U54AG063546, which funds the NIA Imbedded Pragmatic Alzheimer's Disease and AD‐Related Dementias Clinical Trials Collaboratory (NIA IMPACT Collaboratory). The content is solely the responsibility of the authors and does not necessarily represent the official views of the National Institutes of Health. CG, RE, AG, SG, DK, IL, SLS, HM, NM, DMH, RS, CS, CS, MS, ASG have no funding sources to report in relation to this work. Author disclosures are available in the supporting information.

## CONSENT STATEMENT

Consent was not necessary for this article.

## Supporting information



Community Briefing

Supporting Information
