# Supplementary material for: Public and participant involvement as a pathway to inclusive dementia research
Source: Alzheimers Dement. 2024 Nov 14;21(1):e14350. doi: 10.1002/alz.14350 (PMC11782197; doi:10.1002/alz.14350)
Supplement: Supplementary file 1 — Community Briefing [file ALZ-21-e14350-s002.docx]

**Public and Participant Involvement as a Pathway**

**to Inclusive Dementia Research**

Community Briefing

Aug 7, 2024

Research that is inclusive and brings in people from all walks of life is an urgent priority for dementia research. Communities that have been most impacted by dementia do not typically make up their share of people involved in research. Public and Participant Involvement (PPI) is the pathway to science that involves people from these under-engaged communities. PPI can improve every type of research, whether the focus is on health care or social services, or clinical trials testing new medications.

A group of researchers and community members worked together to write a position paper with the goal of helping researchers understand how to best partner with communities.

Before researchers engage in communities, it is important for them to understand the stigma, or negative effects, that older people experience just because of their age. This is much worse for older people that also have a diagnosis of dementia. People with a dementia diagnosis are fighting these negative ideas with a simple and strong message, “I am still here”. The impact is greater for people who have dealt with discrimination for their entire lives because of their skin color, their religion, their gender identity, or abilities. Researchers must take time to first educate themselves. They can begin by asking community members, “What are your needs? What are the biggest issues impacting your health and your community?” These discussions must be bidirectional, and researchers must be prepared to transparently respond to questions. “*Community members may ask of researchers, ‘Why should I trust you? You need to establish a relationship; a dialogue; I getting to know you and you getting to know me’.”*


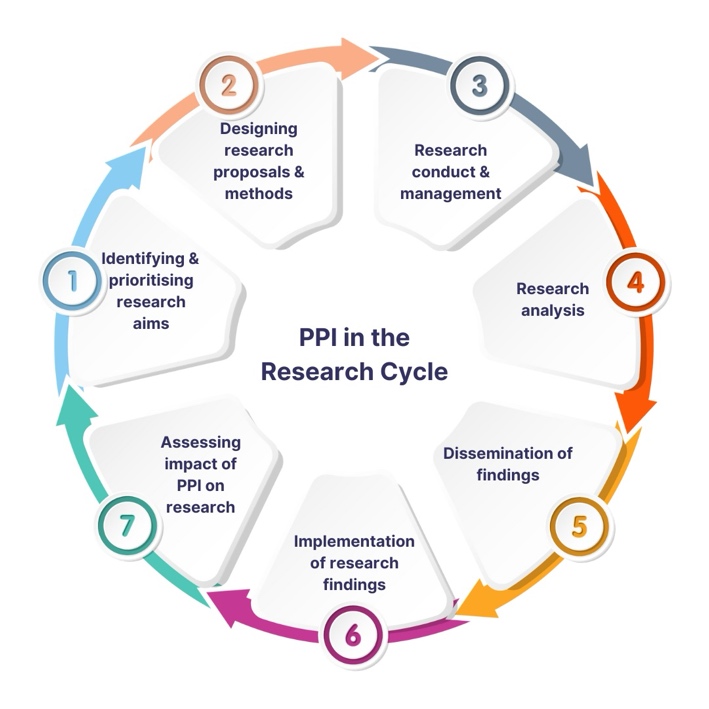
The best partnerships are built by involving communities in every step of research. This is illustrated in the diagram: PPI in the Research Cycle. Detailed examples are provided in the full paper.

In Step 1, Community members can guide researchers to understand what is the most important issues that research can help to address. In Step 2, community members can guide how research is done. This could include deciding how to invite people to research, developing materials together with researchers that describe the study. Steps 3 and 4 involve community members in collecting and making sense of the data. At Step 5, sharing what is learned is an important place for communities to be involved. They can ensure the information reaches everyone and that all communities can benefit from new learnings. Step 6 is another important role, where what is learned from the research results in changes to health care or social care, including policy changes. Finally, Step 7 is where community members help researchers understand how being involved in the research made a difference, for themselves as well as for their community.

We also developed recommendations for how to create and maintain close collaboration between researchers and participants through PPI (see table). There are practical ways that researchers can build trusting relationships with community members. To build truly equal partnerships with communities, researchers must take the time to listen and understand the day-to-day challenges people are dealing with. This is very important for communities that don’t have equal access to health care or social support. Meaningful partnerships are built on choice, respect, shared decision making, access, inclusion, and representation.

In conclusion, inclusive PPI upholds the dignity and rights of study volunteers and offers clear benefits to research. Our shared goal for dementia research is best captured by the perspective of community members. *“We want to see dementia research accelerate and provide meaningful benefits to their lives, the lives of their loved ones, their children, and their communities. We seek to be seen, valued, and treated always as a person first.”*

---This paper and community briefing were developed by a group of researchers and community members as part of the ISTAART PIA: Partnering with Research Participants. Lead author: Sarah Walter, MSc, Alzheimer’s Therapeutic Research Institute, University of Southern California ([waltersa@usc.edu](mailto:waltersa@usc.edu))

Community member co-authors: Sandra Loyola Sandoval, Helen Medsger, Nancy Meserve, Roland Samaroo, Cynthia Sierra, MS, Marlon M P Smeitink, MA BSc

| **Table 2. Recommendations for Public and Participant Involvement (PPI) in ADRD Research** |
| --- |
| **Establish a partnership with choice and shared decision making.** Ground partnership by valuing the needs, lived experience, and expertise of the community participants. |
| **Ensure equal access.** Ask for input on how to make interactions effective and accessible, including how group is structured, and settings for interactions. |
| **Start early, but it’s never too late to engage.** PPI input can influence the focus and priorities of research, so it’s best to start partnerships early in the process of research. |
| **Ensure representation and respect differences.** Be intentional in composing your group, ensuring voices not commonly heard are given space. Avoid single representatives or scenarios where PPI are outnumbered by researchers. |
| **Discuss and define terms, titles, and roles.** The language we use demonstrates partnership, discuss with the community, and avoid acronyms and ‘academic speak’. |
| **Encourage storytelling.** Storytelling is a legitimate method of sharing wisdom, and patient stories are an important piece of evidence to inform care. |
| **Share impact.** Summarize for both community and researchers the impact of PPI on research and involve PPI in dissemination of learnings to both general audiences and scientific spaces. |
| **Demonstrate appreciation.** Ask what incentives would best show appreciation, and would be meaningful and helpful, such as a financial honorarium or a certificate or public acknowledgement, will differ across communities.^85^ |
| **Offer training to support co-learning.** Training can benefit and empower both PPI members and researchers, but make sure training is not a barrier to participation. |
| **Listen and keep listening.** Feedback from PPI will clarify understanding on the part of both researchers and community. |
| **Respect confidentiality and privacy.** Assure PPI that their input will be kept confidential and anonymous. |
| **Demonstrate long-term commitment.** Make long-term plans, work towards short term goals, plan together with community. |
